# Estimating the Radiation Dose to the Fetus in Prophylactic Internal Iliac Artery Balloon Occlusion: Three Cases

**DOI:** 10.1155/2015/170343

**Published:** 2015-06-09

**Authors:** Kentaro Kai, Tomohiro Hamada, Akitoshi Yuge, Hiro Kiyosue, Yoshihiro Nishida, Kaei Nasu, Hisashi Narahara

**Affiliations:** ^1^Department of Obstetrics and Gynecology, Oita University Faculty of Medicine, Oita 879-5593, Japan; ^2^Department of Radiology, Oita University Faculty of Medicine, Oita 879-5593, Japan

## Abstract

*Background*. Although radiation exposure is of great concern to expecting patients, little information is available on the fetal radiation dose associated with prophylactic internal iliac artery balloon occlusion (IIABO). Here we estimated the fetal radiation dose associated with prophylactic IIABO in Caesarean section (CS). *Cases*. We report our experience with the IIABO procedure in three consecutive patients with suspected placenta previa/accreta. Fetal radiation dose measurements were conducted prior to each CS by using an anthropomorphic phantom. Based on the simulated value, we calculated the fetal radiation dose as the absorbed dose. We found that the fetal radiation doses ranged from 12.88 to 31.6 mGy. The fetal radiation dose during the prophylactic IIABOs did not exceed 50 mGy. *Conclusion*. The IIABO procedure could result in a very small increase in the risk of harmful effects to the fetus.

## 1. Introduction

Over the past decade, the procedure known as prophylactic internal iliac artery balloon occlusion (IIABO) for suspected placenta previa/accreta has become more frequently performed against a background of significant associated morbidity, uncertainties in antenatal diagnosis, the desire for fertility preservation, and advanced interventional radiologic techniques [1].

However, little information is available on the fetal radiation dose associated with prophylactic IIABO. The IIABO procedure is performed under fluoroscopic guidance. There are potential risks related to ionizing radiation, and fetal radiation exposure is of great concern to expecting patients because fetal tissues are more susceptible to radiation injury [2]. In view of the possibility of radiation-induced cancer in the fetus [3], a fetal dose estimate and a critical risk-benefit analysis for the mother and the fetus must be conducted prior to the initiation of the IIABO procedure [4].

The purposes of the present study were to (1) estimate the fetal radiation dose associated with prophylactic IIABO during Caesarean section (CS) and (2) report our experience with this procedure in three patients with suspected placenta previa/accreta.

## 2. Case Presentation

Three patients for whom sonographic and MRI findings revealed possible placenta previa/accreta were scheduled to undergo CS deliveries. Because of the high probability of major hemorrhage and the potential need for a cesarean hysterectomy in all three patients, each of the patients was referred to our interventional radiology department for the temporary placement of occlusive balloon catheters in the internal iliac arteries before her CS delivery and possible hysterectomy. We conducted a geometrical estimation to obtain phantom dose measurements as close as possible to the actual values, using the same equipment as that used for the patients, and then we used the simulated values to estimate the radiation dose to the fetus.

### 2.1. Fetal Radiation Dose Simulation

**Figure 1:**
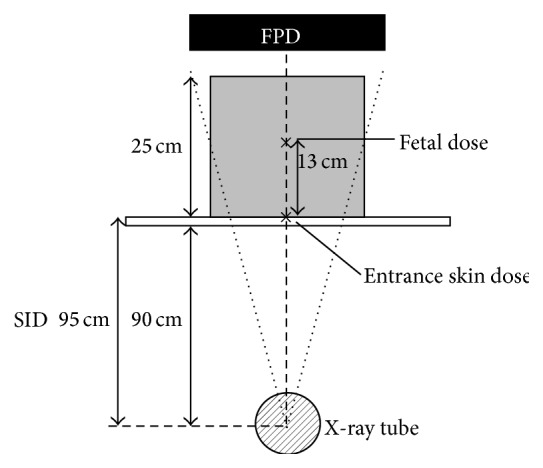
Schematic of the acrylic phantom used to simulate the fetal radiation dose in the angiography suite. FDP, flat-panel detector; SID, source-image distance.

We simulated two scenarios: (1) the patient was catheterized in the angiography suite and then transferred to an operating suite and (2) the patient was catheterized in the operating suite. Fetal radiation dose measurements were obtained using an anthropomorphic phantom made by a 10 mm thick acrylic board (41436-000 XAC-type 1, Kyoto Kagaku, Kyoto, Japan).  Figure 1 is a schematic diagram of the acrylic phantom used to simulate the fetal radiation dose in the angiography suite. To simulate pregnancy in the third trimester, we adjusted the overall thickness of the phantom to 25 cm. Measurements were made on an Infinix Active digital subtraction angiography (DSA) system (Toshiba, Tokyo). We measured the entrance skin dose as the interventional reference point (i.e., the isocenter). We measured the fetal dose using a manufacturer-calibrated skin dose monitor (SDM 104-101 V4.0, McMahon Medical, San Diego, CA) at 13 cm from the isocenter; the fetus was assumed to lie at about the center of the phantom [5]. The 5 cm thick board was located 90 cm from the X-ray tube, and the source-image distance was 95 cm. The simulation results are shown in Table 1. The table's column heading for the fluoroscopy “Mode” was as follows. The “Normal” mode provided high X-ray output and high image quality, and the “Low” mode provided low X-ray output and low image quality. These modes were selectivity used based on the operator's need.

**Figure 2:**
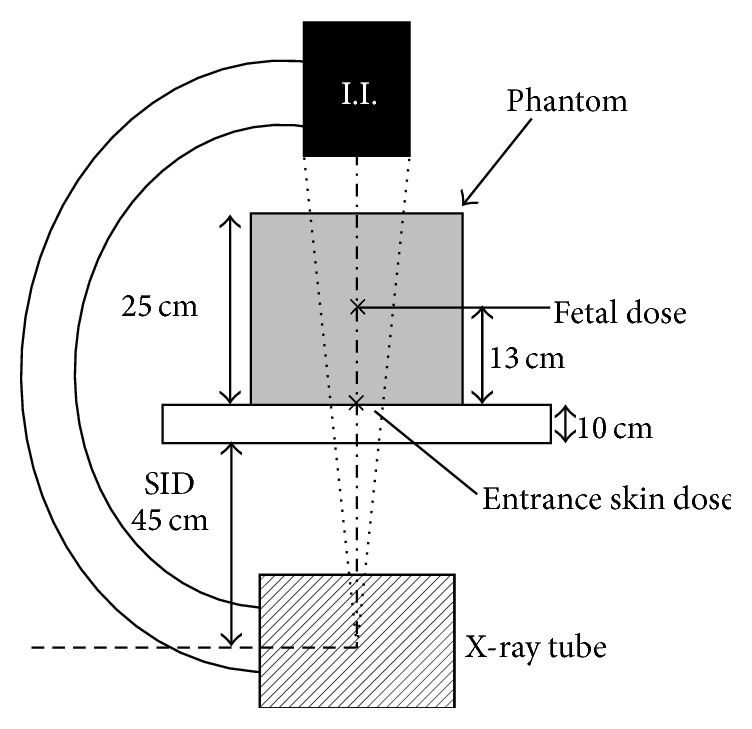
The X-ray tube-image intensifier C-arm in relation to the phantom used to simulate the fetal radiation dose in the operating suite. I.I., image intensifier.

Figure 2 is a schematic of the X-ray tube image intensifier C-arm in relation to the phantom in the operating suite. The irradiation was carried out using a Siremobil Compact L C-arm (Siemens, Erlangen, Germany) or a GE-OEC 9800 C-arm (GE Healthcare, Buckinghamshire, UK) with last-image hold ability. The height of the operating table was leveled with the usual lithotomy position. The X-ray tube voltage and current used with the Siremobil Compact L C-arm were 106 kV and 3.1 mA, respectively. With the GE-OEC 9800 C-arm, the X-ray tube voltage and current were automatically regulated. The simulation results are shown in Tables 2 and 3.

The Institutional Review Board of the Oita University Faculty of Medicine approved this single-center retrospective study, and all three patients provided written informed consent.

### 2.2. Endovascular Technique

Arterial access to both femoral arteries was gained using the percutaneous Seldinger technique under local regional anesthesia with lidocaine. Two occlusion balloon catheters (5-Fr Moiyan; Miyano Medical Instruments, Kobe, Japan) were placed in the most proximal division of the internal iliac arteries through 5-Fr crossover sheaths (Terumo, Tokyo) in the contralateral femoral artery. Sufficient diluted contrast medium (usually approximately 1.0 mL) to occlude the arteries was registered. Fluid flow stagnation with an inflated balloon (dia. 11 mm) was confirmed. Sheaths and balloon catheters were fixed with skin sutures and a drape cover.

Following the cramping of the umbilical cord, the occlusion balloons were blindly inflated. During the entire surgical procedure, the femoral introducer sheaths were flushed with heparinized solution (5,000 IU heparin in 500 mL of saline) using an intermittent bolus technique at 10 min intervals. The balloons were inflated intermittently, after which the CS was performed to confirm adequate hemostasis. The sheaths and balloon catheters were removed immediately after the surgery.

### 2.3. Patients

Table 4 provides information about the patient's surgeries and outcomes. The patients' cases are briefly summarized here.

*Case 1*. A 25-year-old woman was referred to our hospital at 31 wks of pregnancy for suspected placenta previa. She had had two uterine curettages for spontaneous abortion. At 36 wks she underwent a bilateral ureteral stent placement for the prevention of intraoperative ureteral injury during a possible emergency Caesarean hysterectomy. On the morning of the elective CS, bilateral catheterization of the internal iliac arteries was performed in an angiography suite, using the Infinix Active DSA system. After the patient's transfer to the operating suite (scenario #1), we confirmed the position of the balloons using Siremobil Compact L C-arm fluoroscopy. After the cesarean delivery, the sleeping infant needed life support by neonatologists in the neonatal intensive care unit (NICU). The placenta was separated. The final histological examination showed no findings of placenta accreta. The mother and infant left the hospital 10 days after the CS, without complications.

The fluoroscopy with the Infinix Active DSA system in the angiography suite was performed using the condition of middle mode-12 f/s. The subtotal radiation dose in the angiography suite was 19.7 mGy. After the patient's transfer to the operating suite, the position of the balloons needed to be confirmed by Siemens Siremobil Compact C-arm fluoroscopy. The total fetal radiation dose was 31.6 mGy (Table 5). We followed the infant to 4 yrs of age. We detected no congenital defects that could be related to the radiation dose. 

*Case 2*. A 37-year-old woman was evaluated at 28 wks for bleeding and uterine contractions. Nine months earlier, she had undergone debulking surgery—reducing the mass—for adenomyosis, adhesiolysis for pelvic endometriosis, and right uterosacral nerve ablation for chronic pelvic pain. She became pregnant during the third trial of* in vitro* fertilization and embryo transfer (IVF-ET). At 32 wks, considering the recurrence of bleeding and the significant drop in the patient's hemoglobin (from 11.7 to 8.8 gm/dL), an emergency CS was scheduled. Bilateral ureteral stents were placed preoperatively. Immediately after a bilateral catheterization of the internal iliac arteries, the emergency CS was performed with rapid sequence induction. We performed all angiographic and surgical procedures in the operating suite using the GE-OEC C-arm (scenario #2). The placenta separated, but a 3 × 3-cm fragment of the placenta was firmly adherent to the posterior lower segment of the uterus.

Following medical hemostasis with a local injection of diluted Dinoprost, the balloons were deflated. Active hemorrhaging from the retained placenta resumed, and thus the balloons were inflated and four points of surgical hemostasis with #0 absorbable sutures were performed. After hemostasis, the retained placenta was left* in situ*. The sleeping infant needed 7 wks of neonatal intensive care. The mother left the hospital 3 wks after the CS, without complications. The delivered placenta showed no findings of placenta accreta histologically.

The fluoroscopy with the GE-OEC C-arm was performed using the normal mode. The total fetal radiation dose was 18.0 mGy (Table 5). The infant had isolated congenital patent ductus arteriosus, and the initial pharmacologic therapy succeeded in closing it. We followed the infant to 5 yrs of age, and she remained healthy. 

*Case 3*. A 42-year-old woman was transferred to our hospital at 26 wks for uncontrolled bleeding after cervical polypectomy. She had had two uterine curettages for one miscarriage and one first-trimester abortion. She became pregnant during the first trial of IVF-ET after a debulking surgery for adenomyosis. At 37 wks an elective CS was performed with combined epidural and general anesthesia without ureteral stent placement. We performed all angiographic and surgical procedures in the operating suite using the GE-OEC C-arm (scenario #2). The infant needed 2 wks of life support in the NICU. The placenta separated. The final histological examination showed no findings of placenta accreta. The mother left the hospital 2 wks after the CS, without complications.

The fluoroscopy with the GE-OEC C-arm was performed in the low mode. The total fetal radiation dose was 12.88 mGy (Table 5). We followed the infant to 5 yrs of age, and she remained healthy.

## 3. Discussion

Our study produced two observations. First, based on the simulated and practical data, we obtained the fetal radiation doses during prophylactic IIABO. The fetal radiation dose during these three prophylactic IIABO procedures ranged from 12.88 to 31.6 mGy. The average fetal radiation dose in our patients was 21 mGy. Second, we observed that performing the angiographic and surgical procedures at the same site reduced the fetal exposure to radiation.

Among the articles that we reviewed, only three studies [6–8] reported the fetal radiation dose associated with prophylactic IIABO (Table 6). Compared to these reports, our study showed minimal fetal radiation exposure. These results raise the possibility that the angiographic procedures used in the prior studies had an effect on the fetal radiation exposure, since in the prior studies all of the angiographic procedures were performed in the angiographic suite, and then the patients were transferred to an operating suite for the CS.

In their practice guidelines, The American College of Obstetricians and Gynecologists [3] notes that the risks of fetal effects, including childhood cancer induction, are thought to be small at a dose of 100 mGy and to be negligible at doses <50 mGy. The U.S. National Council on Radiation Protection (NCRP) stated that, even for a highly unlikely 50 mSv dose—the X-ray radiation weighting factor of which is 1.0—to a pregnant woman, the risk is small compared to other risks to the fetus [9]. The International Commission on Radiological Protection (ICRP) estimates a 0.06% increased absolute cumulative risk of fetal cancer for each additional 10 mGy after a child aged 0 to 15 years old receives whole-body radiation [10]. Based on the ICRP report, the absolute cumulative risk of cancer among our patients ranged from 0.8 to 1.9%.

Neel et al. [11] reported that in cohorts totaling over six million children across a 40-year period, that is, the children of survivors of the atomic bombings in Hiroshima and Nagasaki and the children of a suitable control population, no statistically significant effects of radiation emerged with respect to malignancies in the subjects up to 20 years old. Although the long-term effects of radiation are not known, given the current evidence, the risk to the fetus from the doses obtained by our simulation appears negligible.

Performing the angiographic and surgical procedures in the same suite reduced the fetal exposure to radiation. Limiting the fluoroscopy time is one of the most important steps to reduce the radiation dose [12]. Case 1, the first case in our institutional experience, was catheterized under fluoroscopic guidance in the angiography suite and then transferred to the operating suite for her CS. Although catheterization in the angiography suite has many benefits with respect to the X-ray output, image quality, and other types of support equipment for interventional radiology, we had to reconfirm the position of the IIABO catheters in the operating suite. The management of Case 1 initiated a change of practice in our unit.

**Figure 3:**
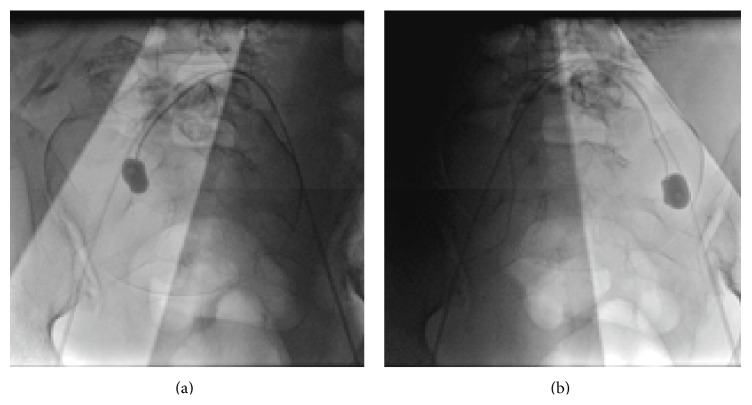
A series of images acquired during the placement of the catheters using shielding. (a) right internal iliac artery and (b) left internal iliac artery.

In Cases 2 and 3, all CS procedures were performed in the operating suite, with the interventional radiologist placing the IIABO catheters using the C-arm, resulting in a lower total radiation dose. In therapeutic radiology, steps should also be taken to minimize the radiation beam so that it affects only the area of interest [13]. We used shielding to ensure minimal fetal radiation exposure during the placement of the internal iliac catheters (Figures 3(a) and 3(b)).

Some limitations of our study warrant consideration. First, we overestimated the risk of placenta accreta in our cohort. There are still uncertainties in the antenatal diagnosis of placenta accreta [14], but preparations for massive peripartum blood loss and extended surgery are an important precaution. Second, the different radiological technique and the presence or absence of adherent placenta had some effect on the fetal radiation dose. The technical differences between first case and the others was the additional fluoroscopy to confirm the position of the balloons, and all fluoroscopic procedures were completed preoperatively. The estimated fetal radiation dose obtained in our case series was a relatively validated measurement.

In conclusion, we identified two important clinical issues. The estimated fetal radiation dose during prophylactic IIABO did not exceed 50 mGy. Performing angiographic and surgical procedures in the operating suite under C-arm fluoroscopy can avoid the need for a reconfirmation of the balloon catheters' position and is a potential approach to reduce fetal exposure to radiation. Although further reports should be accumulated to determine the long-term prognosis of the fetus, the IIABO procedure could result in a very small increase in the risk of harmful effects to the fetus.

## Figures and Tables

**Table 1:** Simulated results of radiation exposure with the Infinix Active DSA system in the angiography suite.

I.I. Size	Mode	Fetus		Skin	
		Dose rate (mGy/min)	DSA^*∗*^ (mGy/f)	Dose rate (mGy/min)	DSA^*∗*^ (mGy/f)
14 inches	Normal	7.0	NA	54.4	NA
	Middle				
	15 f/s	4.6	NA	38.2	NA
	12 f/s	3.6	0.5	30.2	NA
	Low	2.0	NA	17.4	NA

^*∗*^The pulsed fluoroscopy rate was 24 fps with 12 sec image acquisition. I.I., image intensifier; f/s, frame per second; DSA, digital subtraction angiography; NA, not available.

**Table 2:** Simulated results of radiation exposure with the Siemens Siremobil Compact C-arm in the operating suite.

I.I. Size	Mode	Fetus		Skin	
		Dose rate (mGy/min)	DSA^*∗*^ (mGy/f)	Dose rate (mGy/min)	DSA^*∗*^ (mGy/f)
9 inches	Normal	3.4	NA	46	NA

^*∗*^Previously performed in angiography suite, DSA in the operating suite was unplanned in this simulation. Abbreviations are as in Table 1.

**Table 3:** Simulated results of radiation exposure with the GE-OEC C-arm in the operating suite.

I.I. Size	Mode	Fetus		Skin	
		Dose rate (mGy/min)	DSA^*∗*^ (mGy/f)	Dose rate (mGy/min)	DSA^*∗*^ (mGy/f)
12 inches	Normal	5.6	2.4	42.2	22.0
	Low	2.0	1.4	16.6	11.6

^*∗*^The pulsed fluoroscopy rate was 8 fps with 10 sec image acquisition. Abbreviations are as in Table 1.

**Table 4:** Case series.

Case	Parity	Previous CS	Surgical procedure	Anesthesia	Balloon inflated	EBL (mL)	Blood transfusion	ICU	Baby, sex, and weight	Apgar score
1	G2P0	0	El CS at 37 wks	GA	Yes	830	4 units AB	No	Female, 2788 g	2/7

2	G0P0	0	Emg CS at 32 wks	GA	Yes	1315	None	Yes	Female, 2046 g	3/4

3	G2P2	0	El CS at 37 wks	CSE	Yes	1500	6 units AB	No	Female, 2745 g	5/7

G, gravida: P, para: EL CS, elective cesarean section: Emg CS, emergency cesarean section: GA, general anesthesia: CSE, combined spiral-epidural anesthesia: AB, autologous blood.

**Table 5:** Estimated fetal radiation dose in our patients.

Patient number	Fluoroscopy (mGy/min × min)	DSA (mGy/f × times)	Subtotal (mGy)	Total (mGy)
1	3.6 × 5.2 = 18.7^*∗*^	0.5 × 2 = 1.0^*∗*^	19.7	
	3.4 × 3.5 = 11.9^†^		11.9	31.6

2	2.0 × 5.4 = 10.8	2.4 × 3 = 7.2	18.0	18.0

3	2.0 × 5.32 = 10.64	1.4 × 4 = 2.24	12.88	12.88

^*∗*^Angiography suite. ^†^Operating suite.

**Table 6:** Summary of studies of the fetal radiation dose from prophylactic balloon catheterization.

Reference	Year	Patients (*n*)	Occlusion site	Fetal radiation dose (mGy)
Levine et al. [6]	1999	9	Internal iliac (*n* = 7) and uterine (*n* = 2) arteries	61 (median of 9 cases)

Bodner et al. [7]	2006	3	Internal iliac arteries	32 (mean of 3 cases)

Yi et al. [8]	2010	1	Internal iliac arteries	55

This study	2013	3	Internal iliac arteries	21 (mean of 3 cases)
